# Recent advances in the detection of glioblastoma, from imaging-based methods to proteomics and biosensors: A narrative review

**DOI:** 10.1186/s12935-023-02947-1

**Published:** 2023-05-20

**Authors:** Arianaz Hosseini, Hami Ashraf, Fatemeh Rahimi, Iraj Alipourfard, Vahid Alivirdiloo, Behnam Hashemi, Yalda Yazdani, Farhood Ghazi, Majid Eslami, Mahdieh Ameri Shah Reza, Mehdi Dadashpour

**Affiliations:** 1 Razavi Cancer Research Center, Razavi Hospital, Mashhad, Iran; 2 Digestive Diseases Research Institute, Tehran University of Medical Sciences, Tehran, Iran; 3 Division of Clinical Laboratory, Zahra Mardani Azari Children Training, Research and Treatment Center, Tabriz University of Medical Sciences, Tabriz, Iran; 4 Institute of Physical Chemistry, Polish Academy of Science, Warsaw, Poland; 5 Medical Doctor Ramsar Campus, Mazandaran University of Medical Sciences, Ramsar, Iran; 6 Department of Bacteriology, Faculty of Medical Science, Tarbiat Modares University, Tehran, Iran; 7 Immunology Research Center, Tabriz University of Medical Sciences, Tabriz, Iran; 8 Stem Cell Research Center, Tabriz University of Medical Sciences, Tabriz, Iran; 9 Department of Medical Bacteriology and Virology, Semnan University of Medical Sciences, Semnan, Iran; 10 Cellular and Molecular Research Center, Qom University of Medical Sciences, Qom, Iran; 11 Department of Medical Biotechnology, Semnan University of Medical Sciences, Semnan, Iran; 12 Student Research Committee, Semnan University of Medical Sciences, Semnan, Iran

**Keywords:** Glioblastoma (GBM), Proteomics, Biofluids, Nanomaterial, Biosensors

## Abstract

Glioblastoma (GBM) is an aggressive type of cancer that originates in the cells called astrocytes, which support the functioning of nerve cells. It can develop in either the brain or the spinal cord and is also known as glioblastoma multiform. GBM is a highly aggressive cancer that can occur in either the brain or spinal cord. The detection of GBM in biofluids offers potential advantages over current methods for diagnosing and treatment monitoring of glial tumors. Biofluid-based detection of GBM focuses on identifying tumor-specific biomarkers in blood and cerebrospinal fluid. To date, different methods have been used to detect biomarkers of GBM, ranging from various imaging techniques to molecular approaches. Each method has its own strengths and weaknesses. The present review aims to scrutinize multiple diagnostic methods for GBM, with a focus on proteomics methods and biosensors. In other words, this study aims to provide an overview of the most significant research findings based on proteomics and biosensors for the diagnosis of GBM.

## Introduction

Glioblastoma is the most common and aggressive primary malignant brain tumor in adults. In 2016, the World Health Organization (WHO) categorized gliomas into three major types based on histological approaches: astrocytoma, oligodendroglioma, and Ependymomas [1, 2]. The risk factors for GBM are almost indefinable, and the clinical course is usually fatal [3, 4]. Diagnosis is based on histopathological findings, but the evaluation of molecular markers, such as methylation of the *O*6-methylguanyl-DNA methyltransferase (MGMT) promoter and isocitrate dehydrogenase (IDH), as well as broader molecular profiling, can be prognostic [3, 4]. Secondary GBM arises from anaplastic astrocytoma or low-grade diffuse astrocytoma and occur in young patients. They have a low degree of necrosis, are predominantly located in the frontal lobe, and have a significantly improved prognosis compared to primary GBM [5, 6]. Although primary and secondary glioblastoma are almost indistinguishable histologically; they have different genetic and epigenetic profiles [5–7].

Several incomplete and definitive efforts have been made to identify specific associations between GBM and occupational exposure and environmental factors. Ionizing radiation is one of the few identified risk factors that clearly shows an increased risk of developing glioma [8, 9]. Radiation-induced GBM is typically observed years after therapeutic radiation for another disease or tumor has been administered. Other environmental exposures, such as pesticides, PVC, smoking, petroleum refining, and synthetic rubber manufacturing have been inaccurately associated with the development of glioma [10]. The presentation of newly diagnosed GBM patients critically varies depending on the location and size of the tumor and the anatomy of the affected brain [11]. Patients often present with intracranial hypertension symptoms, including localized or progressive neurological defects and headaches [11].

Early diagnostic imaging for GBM may include magnetic resonance imaging (MRI) or computed tomography (CT) [12]. MRI with gadolinium contrast highlights almost all GBMs, revealing irregularly shaped masses with hypointense necrotic centers and dense highlight rings [12]. Necrosis is a hallmark of GBM, and the existence is necessary for brain tumors to be classified as grade IV or GBM by the WHO classification system [12]. Treatment of newly diagnosed GBM requires an interdisciplinary approach. The current standard of care involves maximally safe surgical excision followed by combination radiotherapy with temozolomide (TMZ), an oral alkylating chemotherapy agent, and adjuvant chemotherapy with TMZ. However, comprehensive and complete surgical resection of GBM is difficult, as these tumors are often invasive and located in eloquent areas of the brain, that control movement, language, and sensation. This study aims to confirm recent advances in GBM detection, from imaging techniques to proteomics and biosensors.

## GBM biomarkers and detection methods

Different biomarkers are used for different types of tumors. In GBM, nucleic acids, proteins, small molecules, microRNAs (miRNAs), circulating tumor cells (CTCs), extracellular vesicles, tumor tissues, and body fluids are commonly used [13–15]. Biofluid-based detection of glial tumors offers multiple approaches to improving the quality of life in patients with GBM [16]. Early detection of tumors using screening methods can delay the progression of tumor and increase the likelihood of successful treatment [17]. For example, in the more common malignancies such as breast and colon cancer, early discovery of solid tumors has been established through mammography and colonoscopy screenings, which has become a well-established clinical paradigm [18–23].

The analysis of malignant markers in biofluids was initially recognized in colorectal cancer, where elevated levels of serum carcinoembryonic antigen were detected [24–26]. However, the diagnostic importance of this normal physiological protein is limited, as its levels are not necessarily elevated and high ranges are associated with a variety of other cancers [25, 27]. Timely diagnosis and sensitive treatment monitoring remain major challenges in treating GBM [28]. Clinically, response assessment is primarily based on laboratory tests and magnetic resonance imaging (MRI) [28, 29]. However, both MRI and laboratory tests are insensitive measures of disease status. For example, the lowest reliable resolution detection by MRI is on the order of millimeters [28, 29]. Efforts to take advantage of the powerful imaging capabilities of MRI have led to alternative advances. Advanced MRI techniques such as diffusion-weighted imaging (DWI), dynamic contrast-enhanced perfusion imaging (DSC), and MR spectroscopy (MRS) are theorized to provide physiological information that cannot be obtained by conventional anatomical MRI alone. For example, proton-based MRS (or 1HMRS) provides information about metabolic composition within selected target tissue regions, conceptually similar to an “electronic biopsy”. Comparing the relative concentrations of these metabolites reveals factors that help assess the presence of viable tumors within the sample area, such as B cell membrane turnover and neuronal viability. MRS is more attractive than non-diagnostic techniques as it adds only 15 to 30 min to the traditional MRI technique routinely used in the management of patients with malignant glioma [30, 31]. Therefore, extracellular vesicles (EVs) are a reservoir biomarkers with great potential for assessing glioblastoma tumors in situ [30, 32]. Several molecular markers are still under investigation but are routinely used in GBM patients, including isocitrate dehydrogenase (IDH), *O*6-methylguanine DNA methyltransferase (MGMT), VEGF, and epidermal growth factor receptor (EGFR). In addition, tumor suppressor protein TP53, phosphatases, tensin homolog (PTEN), p16INK4a gene, phospholipid metabolites, cancer stem cells, and most recently, imaging biomarkers have all been extensively validated in clinical settings [33] (Table 1).

**Table 1:** GBM biomarkers and detection methods

#	Biomarker	Methods	Comments	Refs.
1	Solid tumor	MRI	Solid tumors are typically highly aggressive, difficult to treat with complete surgical resection or radiotherapy, and are associated with frequent recurrences and poor prognosis	[34, 35]
2	miRNAs	RT-PCR	Some miRNAs, such as miR-10b, miR-5096, mi-R-709, and miR-19a to contribute to oligodendrocytes’ differentiation	[36, 37]
3	IDH IDH IDH	Miniature mass spectrometer	IDH mutant GBM represents the terminal malignant progression of IDH mutant diffuse astrocytoma (WHO grade II) or IDH mutant anaplastic astrocytoma (WHO grade III)	[38, 39]
		MRI	–	[40]
		Multiparameter MRI	–	[41]
4	EVs	Mass spectrometry	EVs derived from the serum of GBM patients are also associated with tumor-driving cytokines that support the Th2 phenotype rather than the Th1 phenotype	[31, 42]
5	EGFR	Mass spectrometry	Many changes in the EGFR gene have been identified in gliomas, particularly glioblastomas, including amplifications, deletions, and single nucleotide polymorphisms (SNPs)	[43]
6	p^16INK4a^ gene	Gen methylation	p16^INK4A^ is a tumor suppressor gene commonly associated with mutation and/or deletion found in many human tumors, including glioblastomas, melanoma, and leukemias	[44, 45]
7	Phospholipid metabolites	ELISA	Lipid metabolism, particularly phospholipid metabolism, is significantly altered in various types of cancers, including GBM	[46–48]
8	Cancer stem cells	MRI	GBM, the most common and malignant primary brain tumor, contains self-renewing, tumorigenic cancer stem cells (CSCs) that play a role in to tumor development and contribute to resistance to therapy	[49, 50]
9	PTEN	Next generation screening	PTEN is a PIP3 phosphatase that functions as an antagonist to carcinogenic PI3 kinase signaling. It is one of the most potent mutant tumor suppressors, particularly in brain tumors, as it plays an crucial role in suppressing strong signaling pathways	[51, 52]

Diagnostic imaging is one of several techniques for GBM diagnosis, as detailed in Table 1. Despite its benefits, this approach has certain disadvantages, the most significant of which is its lack of specificity. Imaging technology also needs expensive, high-tech equipment as well as qualified employees

*MRI* Magnetic resonance imaging, *RT-PCR* Reverse transcription polymerase chain reaction, *ELISA* Enzyme-linked immunosorbent assay, *TH2* T helper 2, *TH1* T helper 1, *EVs* Extracellular vesicles, *IDH* isocitrate dehydrogenase, *MGMT* O6-methylguanine DNA methyl transferase, *EGFR* epidermal growth factor receptor, *TP53* tumors suppressor protein, *PTEN* phosphatase and tensin homolog

## Proteomics

**Fig. 1:**
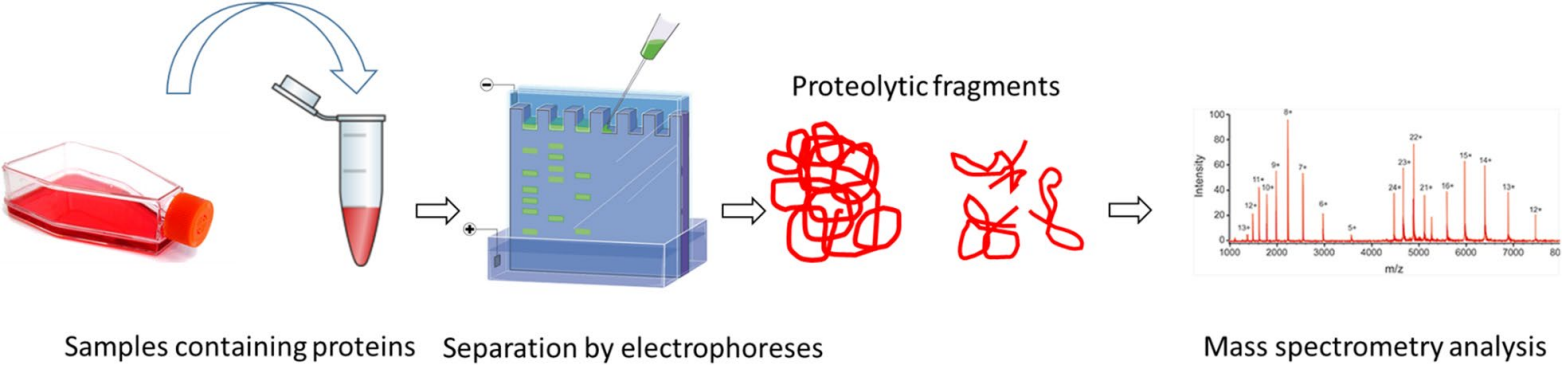
Illustration of proteomics method [56]

Proteomics-based platforms are becoming increasingly powerful in identifying potential disease mechanisms and biomarkers [53]. Proteomics involves using highly complex protein screening techniques for large-scale biological understanding [53]. This information can be combined with genomic data to achieve a better understanding of the underlying biological mechanisms in Guillain–Barré syndrome (GBS) [53, 54]. A typical sample pretreatment method for proteomics analysis is to digest proteins with proteases (such as trypsin or LysC) into peptides, separate them by reversed-phase C18 liquid chromatography, and analyze them using mass spectrometry (LCMS/MS) [55]. Figure 1 for a brief introduction to the proteomics technique.

Proteomics approaches can be readily employed to elucidate the natural production mechanisms of microorganisms and plants [57, 58]. This strategy has also been successfully applied to different types of diseases, such as periodontitis, Alzheimer’s disease, thyroid disease, and various types of tumors [59, 60]. In glioma, proteomics techniques have identified changes in protein expression, but the consistency and biological significance of these changes have not been established [61]. Many innovative proteomic studies are being conducted on several aspects of glioma immunotherapy, including oncolytic viruses, monoclonal antibodies, dendritic cell (DC) vaccines, and chimeric antigen receptor (CAR) T cells [61]. Therefore, the application of proteomics in immunotherapy may accelerate research into GBM treatment [61]. Human proteomic analysis based on clinical blood mass spectrometry (MS) is a powerful tool for studying cancer biomarkers [62]. Numerous clinical trials for GBM using various quantitative approaches have been reported in the last decade. Sequential window acquisition of all theoretical fragment ion spectra mass spectrometry (SWATH-MS) is a novel quantitative method that combines a highly specific data-independent acquisition (DIA) method with a novel targeted data extraction strategy to acquire the resulting fragment ion dataset [62]. SWATH-MS analysis offers several advantages in discovering proteomics, including the high reproducibility and reliable quantitative information [63]. By combining SWATHMS and QTAP analysis, eight candidate biomarkers were discovered in the plasma of GBM patients [64]. Mass spectrometry-based label-free quantitative proteomics has been developed to identify and characterize proteins that are differentially expressed in GBM to gain a better understanding of the interactions and functions that lead to disease states. Advanced identification of upstream regulators provides novel potential therapeutic targets. GBM tumors were analyzed by SDS PAGE fractions with internal DNA markers followed by liquid chromatography–tandem mass spectrometry (MS) [65]. The main challenge in GBM research has been identifying new molecular therapeutic targets and accurate diagnostic/prognostic biomarkers. Many current clinical therapeutic targets for immunotoxins and ligand-directed toxins against high-grade glioma cells (HGG) are surface sialylated glycoproteins [66]. A single-cell surface sialoprotein in human GBM tissue, human astrocytes, fetal and adult human neural progenitor cells (NPCs) was characterized and accurately quantified using a bioorthogonal chemistry reporter (BOCR) strategy combined with label-free quantitative mass spectrometry (LFQMS) were established for characterize and accurately quantify of GBM. This approach comprehensively identifies new biomarkers and therapeutic targets for treating malignant glioma using quantitative sialoglycoprotein proteomics with clinically relevant patient-derived primary glioma cells [66]. Additionally, a simple and sensitive targeted proteomic method was established to quantify membrane and protein transcription factors in the degenerated protein pathways of glioblastoma cells. This method utilized liquid chromatography and mass spectrometry assays to provide high detection sensitivity and quantitative data for prognostic analysis and efficacy testing [67]. The most malignant form of all gliomas is GBM, which is characterized by a poor response to treatment and a high degree of heterogeneity. The subventricular zone (SVZ) is a key site of brain neurogenesis and is rich in neural stem cells. Because GBM tumors are often located near the SVZ, they can be classified as either SVZ− or SVZ+. Tumors that are in close proximity to the SVZ are categorized as SVZ+, while tumors that are distant from SVZ are classified as SVZ−. To gain insight into the increased aggressiveness of SVZ+ tumors, proteomics systems such as LCMS/MS and 2DDIGE were applied to examine possible proteomics changes between the two subtypes. While serum proteomic analysis revealed significant changes in various lipid-carrying and acute phase proteins, tissue proteomic analysis showed significant changes in regulatory proteins, lipid binding, cytoskeleton, chaperones, and cell cycle. These results provide clues to the molecular basis behind the increased aggression of SVZ + GBM tumors and may lead to the identification of rational therapeutic targets for improving the treatment of these highly invasive tumors [68].

Real-time quantitative PCR (qRTPCR) and mass spectrometry (MS) were utilized to identify potential targets for long non-coding RNA (lncRNA) HULC that promote GBM progression [69, 70]. A proteomics-based approach in patient samples was used to identify T-cell target antigens in integrated glioblastoma stem cells. A novel immunogenic protein that frequently induces tumor-specific T-cell responses in GBM patients and is also detected in therapy-resistant, restless and slow-cycling GSCs in vitro was discovered in this study. The stable expression of these T cell targets in primary and recurrent GBM supports their suitability for future clinical applications [71].

The results suggest that proteomics involves the use of highly complex protein screening techniques that can be used for a large-scale biological understanding of GBM. This information can be combined with genomic data to provide a better understanding of the underlying biological mechanisms involved in GBM.

## Biosensor technology

**Fig. 2:**
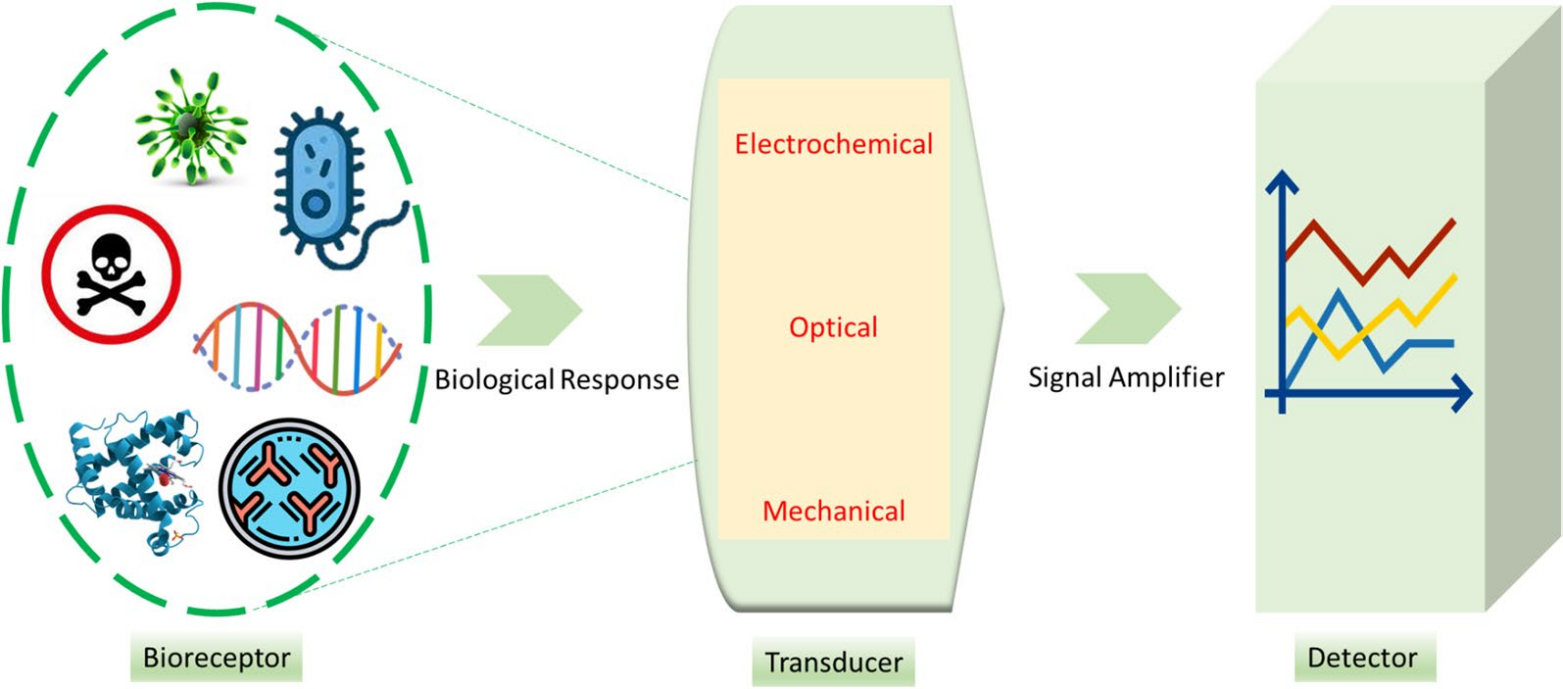
Schematic of biosensor technology

The monitoring and diagnosing various disorders require significant efforts to regularly test blood samples and conduct related tests [72–74]. However, these tests require common analytical techniques, efficient personnel to perform them, and time to collect the necessary samples for clinical trials [75]. Laboratory tests enable qualified personnel to monitor and diagnose a variety of diseases [73, 75]. Specific analytes are known to be specific to a particular disease and may be helpful in monitoring their progression [76, 77]. The clinical usefulness of biochemical tests is determined by their sensitivity to detect disease without false-negative results, and specificity to avoid false positives in individuals who are not ill [76]. Biosensors use the specificity of biomolecules in conjunction with physicochemical transducers to convert biological signals into optical/electrochemical signals [78]. Refer to Fig. 2 for a schematic of biosensor technology.

Numerous metabolite-based biosensors are available to monitor clinically essential parameters such as blood sugar, urea, uric acid, lactic acid, cholesterol, and more [56]. These biosensors are larger than the additional laboratory analysis of substances involved in the clinical analysis [72]. Enzymes are well known for developing biosensors due to their specificity as biological sensor materials and their role in clinical diagnostics has been known for several years. However, enzymes are less stable in solution and need to be immobilized and stabilized for use in biosensor devices [79]. The immobilized phase provides excellent stability and can be reused. Cross-linking, covalent binding, physisorption, encapsulation, and capture are some of the methods used to stabilize enzymes for developing biosensor devices [80]. The matrix or support selected for immobilization depends on the nature of the immobilization method and the biomolecule. Various matrices such as polymer films and carbon, graphite, membranes, gels, LB films, diaphoresis, and conductive polymers have been practiced to immobilize biomolecules/enzymes for developing various types of biosensors [80–82].

### Developed biosensors for the detection of GBS biomarkers

**Fig. 3:**
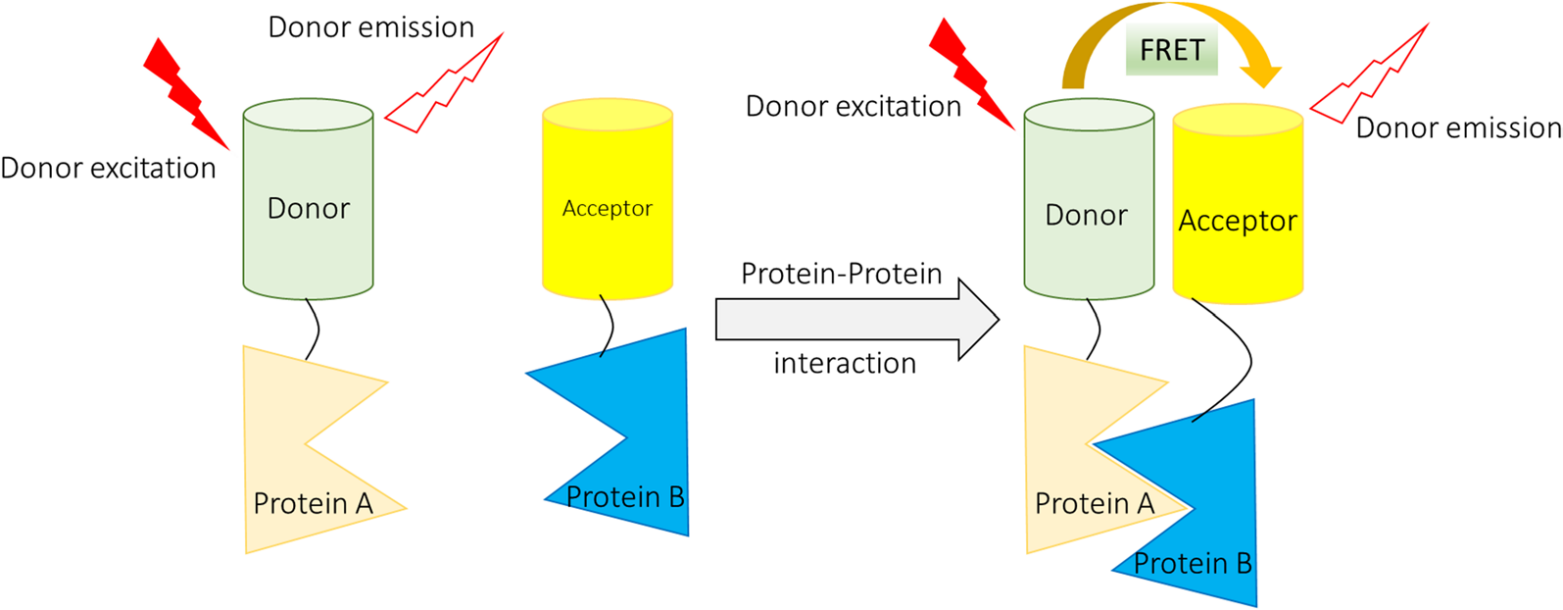
Illustrates the FRET-based approach for detecting protein–protein interactions. Using FRET-based probes, glioma cells that invade the brain parenchyma have higher Rac1 and Cdc42 activity and lower RhoA activity than cells that penetrate the perivascular area. In other words, the FRET-based method was useful for monitoring the invasion mode of GBM cells. This can be controlled by balancing the activity of Rho-family GTPase and Cdc42-specific GEF

This study produced a novel and sensitive electrogenic chemiluminescent (ECL) biosensor system for detecting the p16INK4a gene using a functional paste nanofiber composite-modified screen-printed carbon electrode (SPCE) [83]. Misfolded mutations in the DNA-binding domain of p53 affect its conformation and its ability to bind to chromatin, thereby affecting its ability to regulate target gene expression and cell cycle checkpoint function in many cancers, including GBM. Small molecule drugs that restore the structure and function of misfolded p53 may enhance chemotherapy by activating p53-mediated aging. To determine small molecule-mediated folding changes in the p53 protein a molecular complementation biosensor (NRLUCp53CRLUC) for split renilla-luciferase (RLUC) was constructed. After the initial evaluation of biosensors in three different cell lines, the constructed platform identified the p53P98L mutant endogenously in GBM cells [84]. Fluorescent resonance/Forster energy transfer (FRET) is a non-radiant energy transfer between two molecules that can occur when the two molecules are in close proximity (< 10 nm) [85, 86]. As a result, FRET can be used to measure whether two molecules, such as a ligand and a receptor, interact with each other [85, 86]. For FRET to occur, the fluorescence emission spectrum of the donor must overlap with the absorption spectrum of the acceptor, and the orientations of the transition dipoles must be approximately parallel [85, 86]. Refer to Fig. 3 for an illustration of the FRET-based method in the detection of protein–protein interactions.

**Fig. 4:**
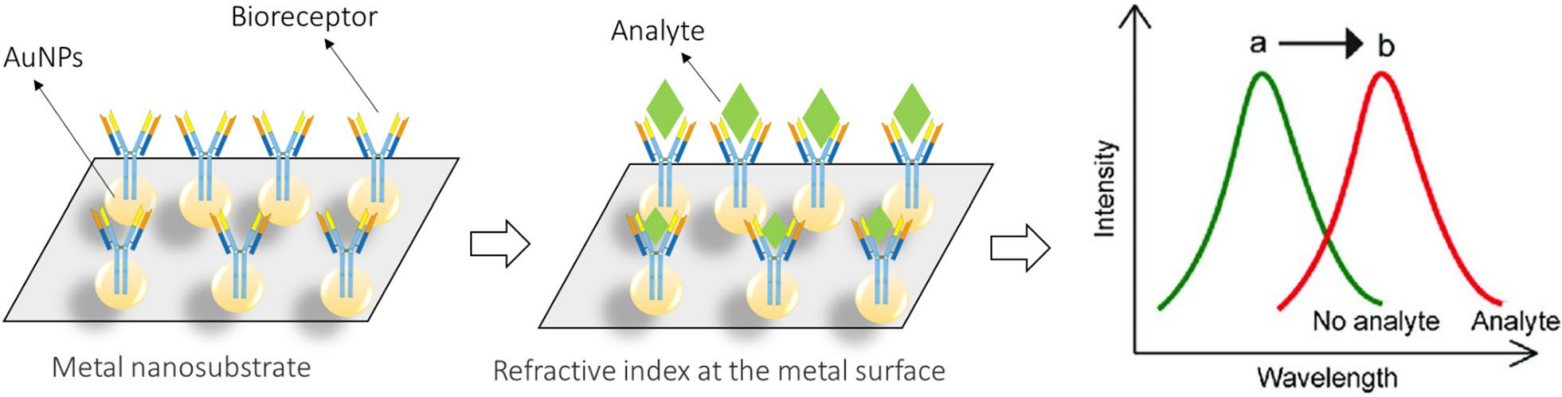
Schematic illustration of LSPR biosensor

Electric cell-substrate impedance sensing (ECIS) is a label-free, real-time impedance-based technique for analyzing cell behavior based on cell adhesion [87]. Several research papers have shown that ECIS is appropriate and can measure GBM cell adhesion. Findings indicated that ECIS reliably measures the adhesion of the differentiated GBM cells on various array types. In addition, ECIS can measure the migration behavior of differentiated GBM cells on the ECIS electrode after alteration [88]. However, GBM stem cells are adhesive, ECIS has a significantly lower ability to measure adhesion compared to differentiated counterparts. This means that while ECIS can be useful for some GBM cultures, it may not be very useful for weakly adherent stem cells [88]. An electrochemical biosensor was developed to detect formaldehyde in aqueous solutions using the enzyme formaldehyde dehydrogenase coupled with a carbon nanotube (CNT) modified screen print electrode (SPE). The proposed system screens the amperometric response to formaldehyde released from U251 human GBM cells in a biosensor compartment in response to treatment with various anticancer prodrugs composed of formaldehyde and butyric acid [74, 89–91]. Surface plasmon polaritons (SPPs), also known as surface plasma waves, are a unique electromagnetic field mode that can appear at the interface between a dielectric and a metal. These SPPs behave almost exactly like a free electron plasma [92, 93]. Surface plasmons are characterized by their propagation constants and magnetic field distribution, and they are in transverse magnetic mode (magnetic vectors are perpendicular to the wave propagation direction and parallel to the interface) [92, 93]. Localized surface plasmon resonance (LSPR)-based biosensing provides a sensitive, unlabeled, inexpensive, and rapid method for detecting biomolecular interactions with nanoscale spatial resolution [94–96]. This technique has promising applications for the robust and sensitive detection of biomolecular interactions. The portability and small size of sensors allow for the miniaturization of sensors to scales not achievable with other planar methods, such as SPR. LSPR-based biosensing devices are easy to manufacture using inexpensive sensing platform. The usefulness of LSPR-based sensing can be enhanced by integrating it into multiplexed microfluidic devices [94, 95]. Refer to Fig. 4 for a schematic of the LSPR biosensor.

**Fig. 5:**
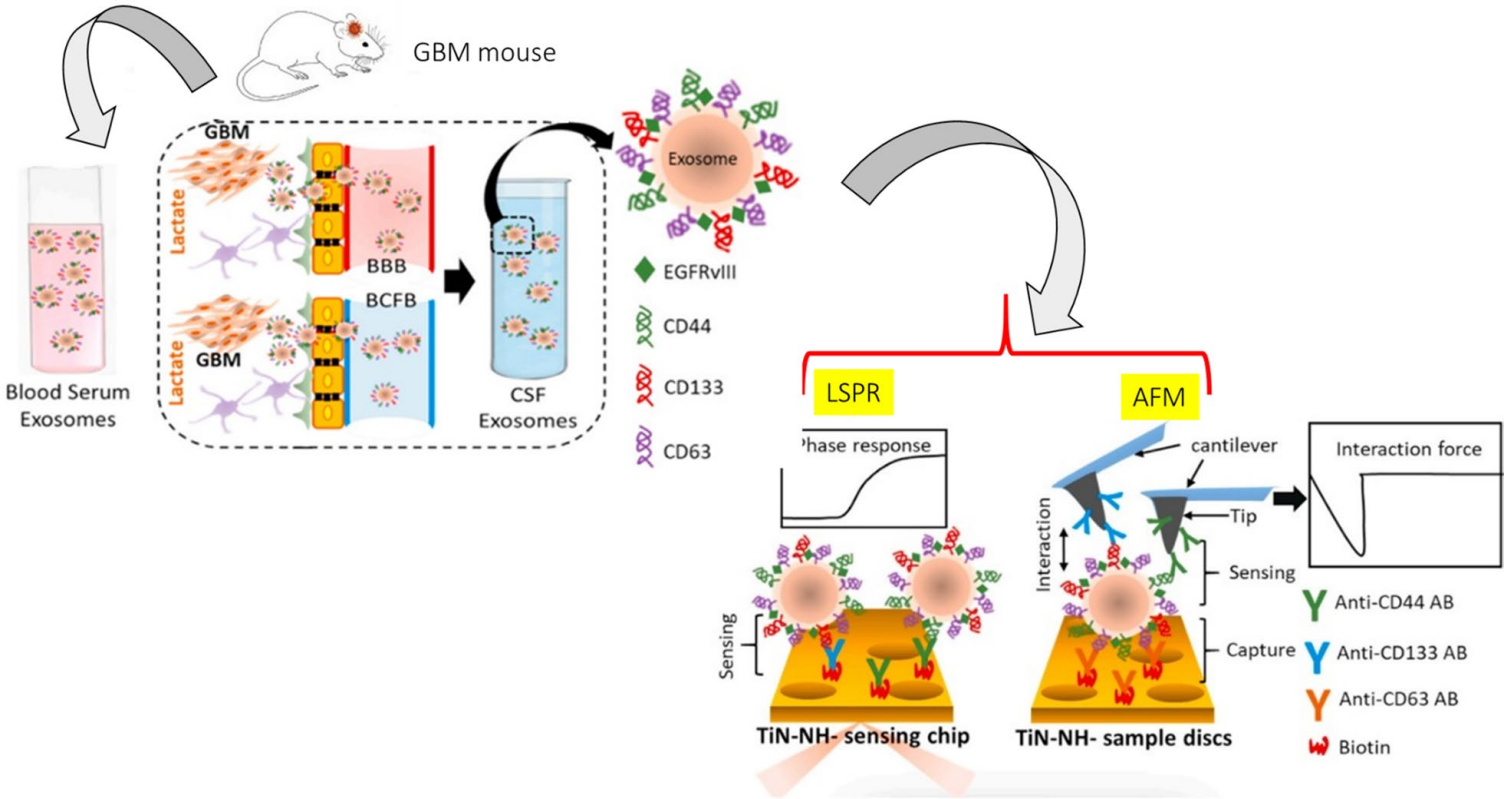
This figure represents a highly sensitive method for detecting exosomal CD44 and CD133 by TIC-AFM and TiN–NH-LSPR biosensors to track the progression of GBM in mice. In the tumor microenvironment (TME), GBM cells utilize accumulated extracellular lactate for their survival, in part by upregulating CD44 and CD133, and releasing exosome. These exosomes not only make the cells more malignant but also enables neighboring ECs to become more angiogenic. This figure is adapted from Ref. [97]

In GBM, secreted lactate promotes the expression of differentiation cluster 44 (CD44) and the release of cell-derived nanovesicles (30–200 nm), such as exosomes, which promotes the malignant progression of tumors. In fatal brain tumors, lactate-driven upregulation of malignant glioblastoma cells (GM) promoted the release of CD44-rich exosomes, increased GM migration and endothelial cell formation, and secreted exosomes. It has been discovered that CD44 can be sensitized by the “capture” of titanium and identifiable by the LSPR biosensor (Refer to Fig. 5) [97].

CDK5 kinase is activated through contact with various partners, including p35/p25 regulators. Active CDK5 plays a vital role in some neural functions, and its hyperactivity contributes to various human cancers and some neurodegenerative processes, especially neuroblastoma and GBM. A probe partner that interacts with CDK5 and a synthetic fluorescent quinolimide-tagged peptide derived from CDK5 calyx were implemented in vitro to detect N2a neuroblastoma and U87 GBM cells [98].

Surface-enhanced resonant Raman scattering (SERRS) is a sensitive and selective method for characterizing bio-molecular sites that exhibit electronic transitions at energies close to or consistent with the laser frequency used [99, 100]. Biomolecules are adsorbed on a suitable metal substrate, usually silver, and measurements are typically performed in situ in a buffer or support medium. This procedure has several advantages. Resonance sensitivity and surface-enhanced Raman scattering (SERS) sensitivity can be combined to enable the use of these methods for measuring nanoscale concentrations [99–101]. The detection of SE(R)RS nanoparticles using Raman spectroscopy-based imaging approach has significant advantages over other molecular contrast agent approaches [102]. For example, compared to fluorescence imaging, our SERRS nanoprobes not only have exhibit superior sensitivity but also have much higher photostability than current fluorochromes [102]. When excited by a single 785 nm excitation source, SERRS nanoparticles with different Raman reporters emits a complex spectrum [102–104]. The ability of integrin-targeted surface-enhanced resonance Raman spectroscopy (SERRS) nanoparticles to accurately depict the true tumor extent in a GBM mouse model that closely mimicking the pathology in humans was appropriately explored. This Raman spectroscopy-based nanoparticle imaging technology shows promise to enabling highly accurate visualization of the true extent of malignant brain tumors [102]. A fluorescent peptide reporter of CDK5 kinase activity derived from a library of CDK5-specific substrates, has been constructed. Its ability to respond to recombinant CDK5/p25 has been recognized and sensitive changes in fluorescence intensity report the CDK5 activity of glioblastoma cell extract. A cell-permeable variant of this biosensor has been further developed, which can be used to map CDK5 activation dynamics spatially and temporally. This offers an exciting opportunity to develop diagnostic assays for neuropathology associated with overactivated CDK5, and companion assays to assess responses to new therapies targeting this kinase [105]. A localized surface plasmon resonance (LSPR) sensor chip was developed to detect infinitesimal amount of exosomal biomarkers. The sensor chip utilized self-assembly silver nanoparticles decorated on gold nano-islands (Ag@AuNIs) sensor chip was used to provide site-specific bio-conjunction of biotinylated antibodies for detecting exosomal surface biomarkers [106, 107]. Additionally, a magnetic covalent organic framework nanospheres-based miRNA biosensor was created for sensitive glioma detection [108] (Table 2).

**Table 2:** Developed biosensors for detection of GBS biomarkers

#	Biomarker	Platform	Technique	NPs	Linear range	Limit of detection (LOD)	Refs.
1	p53	Fluorescence microscopy	Cell imaging	–	0.375 to 250 μM	31.25 μM	[84]
2	Rho-family	FRET	–	–	16,106 cells in 1 ml	20 mM	[109]
3	Stem Cells	ECIS	–	–	–	–	[88]
4	Formaldehyde (AN)	ECL	Amperometric	MWCNTs, SPE	0.1–100 μM	0.1 μM	[89]
5	GBS	LSPR	–	AuNPs	0.005 to 50 μg/ml	5.29 × 10–1 μg/ml	[97]
6	CDK5 kinase	FRET	Uvis spectrum	–	–	0.2 μM	[98]
7	Tumors	SERRS	Molecular Imaging Probe	AuNPs	3.5 nM	10–15 M	[102]
8	CDK5 Kinase	-	Fluorescent	–	10 µg	–	[105]
9	Exosomal MCT4	LSPR	Optical	Ag@AuNIs	0.4 ng/ml	4 × 10^−4^ to 50 μg/ml	[106]
10	miRNA-182	MCOF	EC	Fe_3_O_4_	20 fM	0.1–10 pM	[108]

*p53* Tumor protein p53, *FRET* Fluorescent resonance/Forster energy transfer, *ECIS* Electric cell-substrate impedance sensing, *MCOF* Magnetic covalent organic framework nanospheres, *AuNPs* Gold nanoparticles, *ECL* electrogenic chemiluminescent, *LSPR* Localized surface plasmon resonance, *SERRS* Surface-enhanced resonant Raman scattering, *GBS* Guillain–Barré syndrome, *MWCNT* Multi-walled carbon nanotube, *SPE* Efficiencies of surface

## Comparisons of proteomics and biosensors technology in the identification of GBM

As previously mentioned, there are several ways to diagnose GBM, each with its own advantages and drawbacks. The primary objective of this study is to help guide the selection of an appropriate method for diagnosing GBM. To achieve this goal, this section compares proteomics and biosensing methods. Table 3 summarizes the advantages and disadvantages of proteomics and biosensors in identifying GBM.

**Table 3:** Advantages and disadvantages of proteomics and biosensors in the identification of GBM

Methods	Advantages	Disadvantages
Proteomics	High throughput can evaluate hundreds of polypeptides in a single run and is specifically designed for detecting protein interactions with various molecular types. Antibodies can be used to probe polypeptides and detect post-translational changes. Protein expression can be measured semi-quantitatively, and these techniques are compatible with other methods [110–112]	They only recognizes known proteins and have little dynamic range compared to other isolation techniques. They are also antibody specific and can have difficulty identifying native conformation proteins, signal suppression by extremely abundant proteins can occur, and there is limited repeatability, requiring confirmation for clinical diagnosis. Additionally, proteomics techniques often require small, somewhat pure samples, and making proteins assume their native conformations can be challenging [110–112]
Biosensors	A practical application strategy for a biosensing system should consider several factors, including a wide detection range, low limit of detection (LOD), quick reaction time, low cost and simplicity of the system, good sensitivity and specificity, high selectivity, acceptable stability, and an easy production method [113–115]	The need for large sample sizes, limited sample throughput, a variety of equipment, solution component adsorption on the membrane surface, and effects on charge transfer modes can all contribute to measurement inaccuracy in electrochemical sensing. Additionally, microelectrode surface renewal can be challenging. Therefore, an electrode reactivation protocol that includes complex programmable potential methods may help improve measurement accuracy [113–115]

## Conclusion

Proteomics analysis is a valuable technique in GBM-related research for determining the interrelationships between intracellular proteins. It can provide both qualitative and quantitative information, identify the type of protein expressed, and investigate the phenotype of each expressed protein under different conditions. Similarly, biosensors are potent tools for diagnosing glioma. Validated studies have shown that using biosensors to identify biomarkers in body fluids is appropriate. Other techniques, such as MRI and proteomics, may be suitable for studying tumors, their structure, size, and morphology. In summary, biosensors play an important role in advancing GB sensors for fast, efficient, and inexpensive detection. Continued work and progress in large-scale plasmonic nanostructures is being achieved using various techniques such as microsphere lithography, fabrication of superparamagnetic particles, interference lithography, nanoimprinting, and new designs with improved performance. A more accurate reading method that reduces costs and enables easier testing with large area substrates, along with signal amplification is useful for SPR, LSPR, SEF, SERS, and SEIRA methods.

## Data Availability

Not applicable.
